# A clinical trial alert tool to recruit large patient samples and assess selection bias in general practice research

**DOI:** 10.1186/1471-2288-11-16

**Published:** 2011-02-15

**Authors:** Stephanie Heinemann, Sabine Thüring, Sven Wedeken, Tobias Schäfer, Christa Scheidt-Nave, Mirko Ketterer, Wolfgang Himmel

**Affiliations:** 1 Department of General Practice, University of Göttingen, Göttingen, Germany; 2 Department of Health Sciences, University of Applied Sciences Fulda, Fulda, Germany; 3 Department of Epidemiology and Health Monitoring, Robert Koch-Institute, Berlin, Germany; 4 IT-Choice, Karlsruhe, Germany

## Abstract

**Background:**

Many research projects in general practice face problems when recruiting patients, often resulting in low recruitment rates and an unknown selection bias, thus limiting their value for health services research. The objective of the study is to evaluate the recruitment performance of the practice staff in 25 participating general practices when using a clinical trial alert (CTA) tool.

**Methods:**

The CTA tool was developed for an osteoporosis survey of patients at risk for osteoporosis and fractures. The tool used data from electronic patient records (EPRs) to automatically identify the population at risk (net sample), to apply eligibility criteria, to contact eligible patients, to enrol and survey at least 200 patients per practice. The effects of the CTA intervention were evaluated on the basis of recruitment efficiency and selection bias.

**Results:**

The CTA tool identified a net sample of 16,067 patients (range 162 to 1,316 per practice), of which the practice staff reviewed 5,161 (32%) cases for eligibility. They excluded 3,248 patients and contacted 1,913 patients. Of these, 1,526 patients (range 4 to 202 per practice) were successfully enrolled and surveyed. This made up 9% of the net sample and 80% of the patients contacted. Men and older patients were underrepresented in the study population.

**Conclusion:**

Although the recruitment target was unreachable for most practices, the practice staff in the participating practices used the CTA tool successfully to identify, document and survey a large patient sample. The tool also helped the research team to precisely determine a slight selection bias.

## Background

General practice populations are important sampling frames for health services research, e.g. surveys about disease prevalence, clinical performance and healthcare needs as well as intervention studies and clinical trials [1,2]. Recruiting and surveying patients through their general practitioner (GP) during consultation most closely reflects routine practice and generates evidence from real world settings about important health conditions and related health care problems, thereby increasing external validity [2-4].

The daily demands of a busy practices, however, leave practice personnel (GPs and practice nurses) with little time to make study involvement a priority [5-8]. Even if GPs agree to participate in research studies, many research projects in general practice face problems when recruiting patients [1,5,9-11]. Recruitment is a time-consuming process which encompasses, besides others, identifying possible study participants, applying eligibility criteria, contacting eligible patients and eventually enrolling them in the study. For this reason, clinical trials often restrict the participation of GPs and their staff to the identification and referral of possible patients, leaving the eligibility review and enrolment steps up to external study personnel [10-16]. Such strategies, however, interrupt the recruitment flow, exclude GPs from the research process and may deter less mobile and less communicative patients from study participation.

Under-enrolment or selective enrolment of general practice patients [17-21] can make generalisation of study results inappropriate [22,23]. These restrictions are often not reported in published studies [19] since the direction and magnitude of this potential selection bias is difficult or impossible to estimate due to the lack of data about all possible study participants. The identification of all possible study participants can be automated using standardised search queries on the electronic patient record (EPR). Such automated identification procedures, so-called "clinical trial alert" (CTA) systems, seem to have a favourable effect over more traditional means of participant identification in clinical settings [12,15,24,25].

In order to make high-quality, practice-based research possible, researchers must equip the local practice staff to implement and perform a research project under real practice conditions. In addition, it is necessary for researchers to develop standardized patient recruitment techniques to analyse sample selection bias. In response to these two challenges, we created a CTA-type recruitment infrastructure, which was introduced as an intervention within the framework of an osteoporosis project. This CTA-tool runs in the background of the practice software system and automatically identifies all possible study participants. A standardized computer-based study protocol and computer survey made it possible for the general practice staff to perform all recruitment tasks under real practice conditions and at the same time, transfer data to the study centre about sample selection bias.

The aim of this study was to evaluate the recruitment performance of the practice staff when using the CTA tool according to 4 criteria. The CTA infrastructure should help local practice staff members to realise (1) similar recruitment rates in all GP practices, (2) a set target of 200 enrolled patients per practice, (3) low participant loss between contact and enrolment and (4) low selection bias between identified and enrolled patients.

## Methods

### Setting and research context

In Germany, GPs are required to use EPRs for the documentation of patient treatment in order to receive payment for services. However, there is a great variety of software systems used in German general practices [26].

The CTA tool under study was developed as part of an osteoporosis project in general practice with the aim of surveying older patients to provide a systematic fragility fracture risk assessment. The project includes: (1) automated eligibility identification using the GP's software system; (2) documentation of exclusions and refusals; (3) computer-assisted, standardised baseline risk assessment and 6-month telephone follow-up for incident fractures; (4) automated feedback generation about individual risk factor profiles; and (5) online real-time submission of pseudonymised data to the central study centre (University of Göttingen, Department of General Practice and Family Medicine). An external software company (IT-Choice) programmed the study software for the most commonly used practice software systems.

Osteoporosis has been recognised as an important health problem by the majority of German GPs [27]. The present osteoporosis project was conceptualised as a prospective observational study with a limited number of participating practices. General practices in loosely connected general practice research networks across Germany as well as members of the German College of General Practice and Family Medicine (DEGAM) were invited to participate in the project. Initial contact to GPs belonging to local GP network organisations was made through local network administrators. In addition, individual GPs belonging to the DEGAM were contacted via email using a distribution list provided by DEGAM. All participating general practices were required to have Internet access. The practice staff received financial incentives for their willingness to take part in the study: practice physicians 150 €, practice nurses 50 €. In addition, practice nurses received 10 € per surveyed patient.

This project is embedded in a large, ongoing government-funded primary health care research project (MedViP; Medizinische Versorgung [medical care] in der Praxis), coordinated by the Department of General Practice at the University of Göttingen and approved by the local ethics committee [28]. All details, including data management of the CTA tool, were approved by the ethics committee and discussed in detail with the data security officer of the medical faculty. All participants of the osteoporosis fracture risk survey provided informed consent prior to participation.

### Software development and implementation

A specialized logistical approach and technical infrastructure were developed in close cooperation with epidemiologists, GPs and IT experts. This cooperation was necessary to integrate the clinical study into the CTA system. The result is not just an "alert", but a clinical trial tool, which initiates the entire research process, which can be completed for an individual patient within minutes of its activation. Between May 2007 and November 2007, the study software was implemented in participating practices' software systems consecutively. Each practice used the clinical trial tool to recruit and survey patients for 12 months.

The tool runs on all Microsoft Windows NT based platforms (NT, 2000, XP, Vista, 7). It is set up as an independent background task and monitors the EPRs user interface for the patient data currently displayed on the screen. The data is not stored unless the tool identifies a potential study participant. The collected data is automatically pseudonymised by replacing the internal patient ID with a unique study-ID that cannot be traced back to the internal patient ID. The pseudonymised dataset is transmitted to the study database via a secured and encrypted internet connection (SSL).

Before implementation of the CTA tool, practice personnel from each participating practice received an on-site 1-hour training session. Trainers (MK, ST, SW, TS) visited the practice and explained both the general features and handling of the study software as well as the project-specific tasks, such as obtaining the patient's informed consent or documenting refusal or exclusion criteria. Throughout the entire study period, the research centre provided a direct support hotline.

### Electronic recruitment process

In clinical studies, there are at least four basic recruitment steps. First, the population at risk (net sample) must be identified. Second, eligibility criteria must be applied to the net sample. Third, eligible patients must be contacted for participation. Fourth, consenting patients need to be enrolled. The implementation of these four steps into the CTA-tool are described in the following; potential benefits are listed in Table 1.

**Table 1:** The implementation of four recruitment steps in the CTA tool and possible benefits

Step	Definition	Example	Benefit
Identification	All possible study participants are recognized	Clinical trial alert (CTA) automated recognition running in the background of the practice computer software to filter out all women ≥60 and men ≥70	No patients are missed Net sample automatically documented No effort required for the practice staff

Eligibility check	Specifically defined inclusion and exclusion criteria are applied to the net sample	Practice staff (nurse or doctor) reviews patient data for exclusion criteria and checks appropriate box on screen	Practice staff can document exclusion criteria on the practice computer by making one click

Contact	Eligible participants are informed about the study and are asked to participate	Practice staff introduces the study and asks for patient consent Patient response is documented using practice computer	Practice staff can easily document all patient contacts using the practice computer Study centre can monitor activity

Enrolment	Patients follow through on their intention to participate in the study	Baseline osteoporosis survey is filled in using the practice computer Pseudonymised data set is immediately transferred to the study centre	No patients lost between the GP practice and study centre All survey data is immediately available

#### (1) Identification

**Figure 1:**
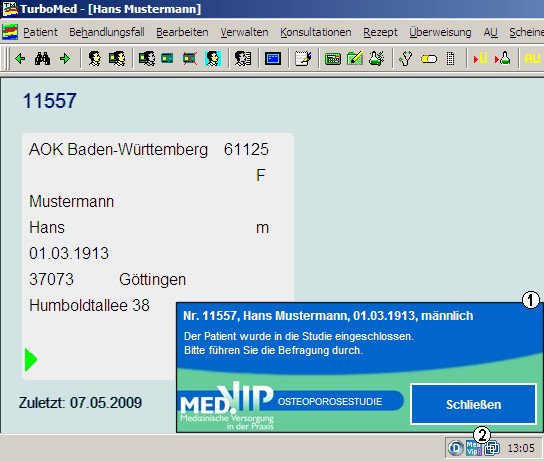
**The clinical trial tool at work**. The EPR for patient "Hans Mustermann" (alias) has been opened. Since Hans is a male over 70, the notification window (1) at the bottom right scrolls up from the notification area of the task bar. The program icon (2) can be seen whenever the clinical trial tool is active.

The CTA tool used patient data (birth date and sex) from the practice software to automatically identify all patients who met the criteria for inclusion into the study. The net sample or population at risk in our study included all women 60 years and older and all men 70 years and older who consulted their GP during the 12-month study period. Whenever a GP or practice nurse opened the EPR of a potential study participant, two things happened simultaneously: First, a pop-up screen on the practice computer reminded the practice personnel that the patient should be considered as a possible study participant, as shown at the bottom right-hand corner of the Figure 1. Second, the study centre received an pseudonymised data set (birth date, sex and the unique study-id). The practice personnel could then either proceed with the next recruitment steps during routine practice or ignore and click off the on-screen reminder.

Even if the practice staff ignored the reminder once, it reappeared on screen whenever the same patient's EPR was opened during the study period. This process continued until further steps of the recruitment process (exclusion, refusal or enrolment) were completed.

#### (2) Eligibility review

If the practice staff member did not click off the on-screen reminder, he or she could continue the recruitment process by reviewing the exclusion criteria. Several exclusion criteria were defined, such as psychiatric disorders, inability to communicate in German or house-bound patients, whose EPRs were opened although they were not physically present in the practice. When a patient's exclusion was documented, the recruitment process finalised automatically and the dataset was sent to the study centre's database.

#### (3) Contact

The practice staff made contact with eligible patients about the study during regular visits to their GP. Staff members informed eligible patients about the content and procedures of the study. If a patient refused to participate in the study, this refusal was documented and his or her anonymous data were transferred to the study centre. If a patient agreed to participate, he or she was immediately enrolled into the study.

#### (4) Enrolment

After agreeing to participate in the study, the practice staff member asked the patient for informed consent. Once consent was documented, an online questionnaire opened and the clinical survey was immediately performed, using a computer-assisted, standardised baseline risk assessment questionnaire. It was possible for the practice staff to interrupt the questioning process at any point and to continue it at a later date.

Throughout all steps of the recruitment process described above, the study centre received pseudonymised data over a secure Internet connection. Patient enrolment was conducted from May 2007 to November 2008. We considered it reasonable to survey, on average, 1 patient per workday and asked the practice staff to aim for a target of 200 enrolled patients.

### Data transfer and data quality

All data transmitted from the participating general practices to the study centre were stored in a relational database (SQL). Patient data sets were pseudonymised through individual code identifiers that could only be re-identified by the practice staff in the local GP practices.

In order to assure that the clinical trial tool worked correctly, we compared the results of its automated, pseudonymised patient registry with complete database reviews of the practice software in a subset of four participating practices. SQL 'filter queries' were computed to identify the number of eligible patients who consulted the practice within the study period. This number of eligible patients was compared to the number of eligible patients recorded by the CTA tool.

### Study design and data analysis

This is an observational study, which looks at the activities of the practice staff (i.e. the study subjects), after the implementation of a CTA-type intervention. The effects of the CTA intervention were evaluated on the basis of four criteria:

1. Patient enrolment in the participating practices, compared with the number of patients identified by the CTA-tool as possible study participants

2. Achievement of the recruitment target (200 enrolees per practice)

3. Amount of participant loss between contact and enrolment

4. Selection bias, determined as the difference in the age/sex ratio between identified and enrolled patients

Throughout the paper, we report absolute numbers and proportions. Since this is a pilot study, we did not perform any power calculations. Differences between the net sample and the enrolees with regard to age and sex were analysed by chi^2^-tests.

## Results

### Participating practices

Of 490 general practices invited for participation, 67 (13.7%) were interested in the study and wanted more information. Among this group of interested GPs, 9 did not communicate further, 5 reported to have no time, 10 did not have an adequate Internet connection in their practice and 16 had to be excluded because the clinical trial tool was not yet programmed for the software systems used in these particular practices.

Finally, 27 practices gave written informed consent to participate. Unfortunately, two practices had to be excluded later from the analysis because the clinical trial tool worked less than 50% of the study period due to problems with the practice computer software. Although data collection in the form of patient surveys continued on in these practices, we have little or no data about the net population, which is vital for an analysis of recruitment efficiency and selection bias. Thus, the analysis presented here is based on data from 25 practices.

Of the 25 participating practices, 14 were single-handed practices and 11 were practices with more than one physician. A total of 27 GPs (7 female, 20 male, mean age: 52 years) and 35 practice nurses took part in the study training.

### Feasibility and precision of the CTA tool

The CTA tool was programmed for, and worked with, the 9 most commonly used practice software systems in Germany. It was implemented consecutively in the 25 participating practices between May 2007 and November 2007. There were notable "downtime" periods in some practices. During these periods, the software could neither collect data nor connect to the database. Mandatory practice software updates, released quarterly by most of the EPR-companies, required the technical support team to make ongoing adjustments and modifications throughout the study period.

The consistency of patient datasets recorded by the CTA tool compared to datasets from a sample of practice software system filter queries in four participating practices showed an average of 91% overlap (ranging from 74% in one practice to nearly 98% in the others).

### Recruitment process

**Figure 2:**
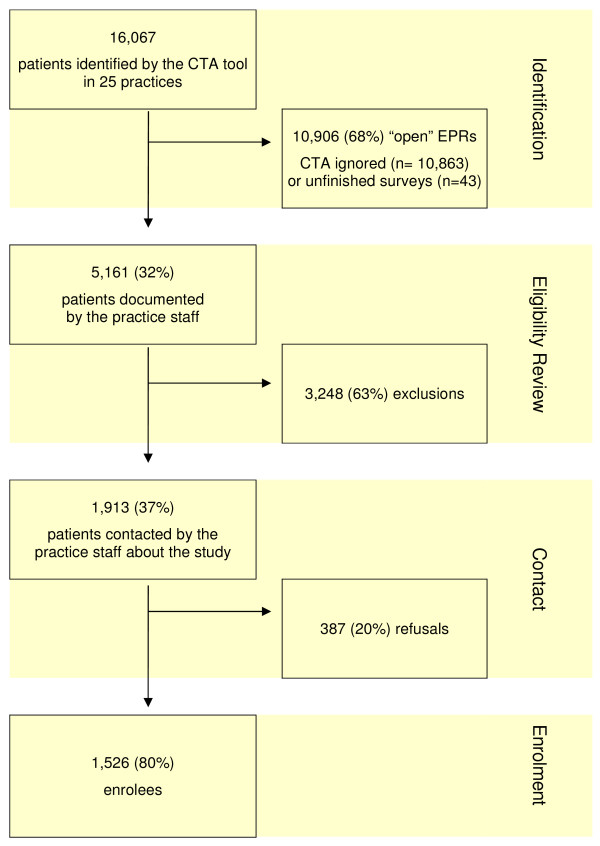
**Patient recruitment: identification, eligibility review, contact and enrolment**.

The CTA tool identified a total of 16,067 potential participants (net sample), with an average of 643 per practice (range 162 to 1,316). In nearly 70% of cases (n = 10,906), the practice personnel did not respond to the CTA, but rather "clicked off" the pop-up reminder. Ignored reminders and unfinished surveys were regarded as being "open" (see Figure 2). In more than 20% (n = 3,248) of cases, the practice staff excluded CTA-identified patients based on set eligibility criteria. A total of 1,913 (12%) patients were contacted by the practice staff about participating in the risk factor survey. Of these, 387 (20%) patients refused to participate. In addition, staff members opened 22 (<1%) EPRs recognised by the CTA tool even though the patients were already deceased. Table 2 shows the recruitment in all 25 practices. In total, the practice staff of the participating practices surveyed 1,526 patients, on average 61 (range 4 to 202) per practice. This corresponds to 9% (range 1% to 26%) of the net sample identified by the CTA tool.

**Table 2:** Enrolment rates and target achievement

	Net sample*	Enrolment		Target achievement***
			
Practice	N	N	(%)**	%
1	162	14	(8.6)	7.0
2	255	31	(12.2)	15.5
3	256	30	(11.7)	15.0
4	334	87	(26.0)	43.5
5	350	82	(23.4)	41.0
6	386	71	(18.4)	35.5
7	390	41	(10.5)	20.5
8	407	53	(13.0)	26.5
9	454	115	(25.3)	57.5
10	455	93	(20.4)	46.5
11	505	4	(0.8)	2.0
12	511	78	(15.3)	39.0
13	596	54	(9.1)	27.0
14	616	99	(16.1)	49.5
15	636	26	(4.1)	13.0
16	689	11	(1.6)	5.5
17	801	133	(16.6)	66.5
18	808	31	(3.8)	15.5
19	901	41	(4.6)	20.5
20	905	9	(1.0)	4.5
21	986	202	(20.5)	101.0
22	1,021	54	(5.3)	27.0
23	1,150	85	(7.4)	42.5
24	1,177	55	(4.7)	27.5
25	1,316	27	(2.1)	13.5
**All**	**16,067**	**1,526**	**(9.5)**	**30.5**

* as recognised by the identifaction and recruitment tool

** in % of the net sample

*** of N = 200 (= target)

In 13 smaller practices (<600 CTA-identified EPRs), the CTA tool identified fewer patients (5,061 vs. 11,006) than the 12 larger practices, but the practice staff was able to enrol a similar amount of study participants (753 vs. 773). Smaller practices recruited, on average, 15% of the population at risk (net sample), compared to 7% in larger practices. Poor recruitment (<5% of CTA-identified EPRs) was more common in large practices (n = 7) than small practices (n = 1).

### Target achievement

Only one practice (practice 21) reached the target to enrol 200 patients within the 12-month study period (Table 2). In total, 31% of the target enrolment (200 patients per practice = 5,000 patients) was achieved (range 2% to 101%). Target achievement for small practices (29%) and large practices (32%) was very similar, in spite of a large discrepancy in the number of CTA-identified EPRs (5,061 vs. 11,006).

### Participant loss between contact and enrolment

All surveys were administered by the practice staff and completed on site in the local GPs practice. In 43 of 1569 cases (3%), the practice staff began surveys without completing them.

### Selection effects

Compared to the CTA-identified EPRs, the practice staff enrolled fewer older persons and men into the study. While 38% of women and 44% of men in the net sample were 80 years and older, these groups contributed only 21% (women) and 29% (men), respectively, to the enrolled sample (see Table 2). Men were generally underrepresented in the study population. They made up 28% of the net sample, but only 19% in the enrolled sample (table 3).

**Table 3:** Study population by sex and age

	Net sample	Not contacted		Contacted	
			
	%	"open" %	exclusions %	refusals %	enrolees %
Gender*	(n = 16,067)	(n = 10,906)	(n = 3 248)	(n = 387)	(n = 1,526)
Women	72.0	70.5	73.2	69.0	81.3
Men	28.0	29.5	26.8	31.0	18.7

Women**	(n = 11,574)	(n = 7,690)	(n = 2,377)	(n = 267)	(n = 1,240)
60 - 69	29.2	31.7	16.4	32.6	37.6
70 - 79	32.4	32.8	25.7	37.1	41.9
80+	38.4	35.6	57.9	30.3	20.6

Men***	(n = 4,493)	(n = 3,216)	(n = 871)	(n = 120)	(n = 286)
70 - 79	56.4	41.5	44.2	56.7	70.6
80+	43.6	58.5	55.8	43.3	29.4

* Chi^2 ^= 80.9; df = 3; p < .0001

** Chi^2 ^= 607.8; df = 6; p < .0001

*** Chi^2 ^= 97.9; df = 3; p < .0001

Ethical approval was granted by the Research Ethics committee of the University of Göttingen Medical School.

## Discussion

This intervention study describes and evaluates the implementation of a self-developed, practice software-based CTA tool for a research project in primary care practices. The study focuses upon the recruitment efficiency of practice staff members who are alerted on their practice computer screen as soon as they open the EPR of a possible study participant. To the best of our knowledge, this is the first study to implement and evaluate such a tool in a primary care setting in Germany. Worldwide, only few trials have reported the successful implementation of similar electronic tools [14,15]. While the CTA infrastructure helped to correctly identify a large population at risk with a minimal workload for the practice staff, the staff in nearly all practices missed the target to enrol 200 patients. However, the enrolment efficiency was high for those patients that were contacted by the practice staff, since the continuous reminder function allowed the practice staff to enrol patients when time and circumstances permitted. The enrolled sample was somewhat biased, but the real-time monitoring of the recruitment process helped the study centre to detect selection bias, since it was possible to track and quantify patient identification and enrolment.

### Limitations

Patients whose EPRs were called up in the practice software during the recruitment period of the study were identified. Unfortunately, this does not completely coincide with the physical presence of patients in the practice during the study, since naturally EPRs are opened when information is needed and the patient may not be in the practice. Therefore, the CTA identified net sample is likely to be larger than the actual number of possible participants. As a consequence, further development of the clinical trial tool will include a documentation option to specify whether a patient is physically present or not.

The only requirements for practice staff participation in the study were willingness, an Internet connection and the use of one of the 9 most common practice software systems in Germany. We made no selection of practices according to size, patient volume, number of staff members or other criteria. We consider it advantageous, not disadvantageous, that the participating practices were heterogeneous (as GP practices in Germany are). Due to the limited number in this pilot study, the results cannot be generalised for general practice recruitment in Germany. In addition, it is not possible to draw any conclusions about the source of selection bias in the patient sample, even though we have been able to detect and quantify it using the CTA tool.

We had no control group of practices without the clinical trial tool. Therefore, we do not know how the implemented recruitment infrastructure and especially the pop-up reminder influenced recruitment behaviour and whether or not an EPR-based recruitment system is more effective than more traditional recruiting strategies in general practice. However, we know from Embi's and Rollman's prior research that EPR-based CTA systems are superior to traditional recruiting methods [12,15]. Moreover, the contact and enrolment rates, realized by the practice staff are rather impressive and may allow valid conclusions for the osteoporosis project.

### Effects and advantages of the intervention

There are some reports in the literature of CTA systems embedded in hospital information systems, outpatient clinics or community health centres to support physicians and nurses in patient recruitment [12,25,29,30]. Our system is the first to support the practice staff in both identifying and surveying eligible patients during daily practice while recording pseudonymised data about the target population in order to monitor selection bias. In a study conducted in the Netherlands [14], the research team used an interactive reminder tool similar to the one described here to inform the GP when an eligible patient was selected by EPR and should be asked to participate in the study. However, eligible patients needed to be selected and marked in the EPR prior to study begin. Another study [16] with a similar technology, used filter queries to generate a list of eligible patients before study begin and provided GPs with a printed version of the list from which to pursue recruitment. In contrast to these identification procedures, the CTA tool described here operates in the background of the practice software. Therefore, a pre-selection procedure is not necessary and even newly inscribed patients matching the inclusion criteria for the study can be reliably identified.

The most important question of our study refers to the efficiency of the CTA tool used in this study. Especially 4 aspects seem to be important:

(1) The practice staff in most practices could identify an ample number of possible study participants for the specific research project (men ≥ 70, women ≥ 60). An average of 643 patients per practice met the inclusion parameters of the study. Although the registration of such a large number of patients seems to be heavy burden for the practice staff, it is noteworthy that this important step of the recruitment process, i.e. identification of the population risk, happened automatically and did not require the practice staff to exert any effort other than clicking off the reminder screen.

(2) Only one practice enrolled 200 patients into the osteoporosis survey. More importantly, many practices, especially larger practices, were poor recruiters although the CTA tool presented these practices a wealth of possible study participants to enrol. Obviously, the regular and frequent presentation of possible study participants on the screen did not stimulate the practice staff to contact them and to start the survey, but had the opposite effect. One reason may be that the clinical trial alert simply appeared too often (e.g. in practices which identified more than 1,000 patients). When the practice staff is frequently reminded about the study, the activity of disengaging or "clicking off" the reminder screen may then consume the majority of the staff's available capacity for participating in the study.

(3) When the contact level is used as the point of reference, the practice staff was able to enrol 80% of the individuals they approached about the study, which we believe to be an efficient use of local general practice staffs' limited capacities for taking part in research projects. The fact that the practice staff only personally contacted 12% of the net sample indicates that the workload in terms of the absolute number of identified patients was too high, which bears the risk of selection bias (see below).

(4) In 5,161 cases, the practice staff invested some effort in the study, i.e. opening the survey and documenting a patient's file. Of these patients, 1,526 (30%) could be enrolled into the study. In comparison, Rollman reported that only 22% of the CTA-identified patients referred from the primary care practice to an external study centre were later enrolled into the clinical study [15]. In Embi's study [12], the CTA intervention increased the referral rate to an external study centre tenfold in comparison to other means of identifying the population at risk. However, the enrolment rate only doubled. In our study, the practice staff completed the osteoporosis survey for nearly all patients that agreed to take part in the survey (1,526 of 1,569; 98%). The combination of a CTA-based identification tool with a practiced-based research design made it possible for the practice staff to survey a large number of patients on site without involving an external study centre.

### Selection effects

One important feature of the CTA tool was the unselected presentation of all patients from the target sample as potential participants of the osteoporosis project. The CTA tool used in our study made it possible to receive real-time information about the recruitment process as well as information about age and sex of all eligible patients in the participating practices; such information is not normally available to researchers. On the basis of this information, it was possible to detect a selection bias. Older patients (80+ years) of both sexes were underrepresented in our surveys, as well as men in general. Therefore, it was possible to quantify the selection bias in terms of age and sex. Choosing and recruiting a representative, non-biased selection of participants or at least estimating the selection bias is a challenge for primary care-based research as it is essential for any generalisability of the study results [3].

In addition to a quantifiable selection bias by demographic variables, the practice staff's limited use of CTA reminders may indicate that enrolees were selected according to other--as yet unknown and undocumented--criteria. The very low enrolment rate (<5%) in some practices may be a sign that the practice staff selected patients according to criteria outside the study protocol. For example, in the context of the present study, it might have been easier to recruit patients who are well-educated and/or communicative, patients who visit the practice during "slow" hours (i.e. disease management afternoons) or patients for whom osteoporosis is an important issue. To become aware of this dimension of selection bias is very important for further research, because clinical studies are built upon the assumption that the individuals who implement the sampling strategy are following the study protocol, i.e. that a random sample of a general practice population is truly random.

### Future challenges and research

The fact that only one practice was able to survey 200 patients within the 12-month study period and that many large practices were poor recruiters will be a stimulus for us to consider the design of future projects. It would have been possible to program the CTA tool to randomly select one patient per workday and require the practice staff to survey this patient. However, past studies in general practices have shown that a rigid study protocol is very difficult for busy general practices to fulfil [22]. Therefore, we programmed the CTA tool to over-supply the staff with possible study candidates, assuming that during the workday, one of these possibilities would pop up at a time that was convenient both for the practice staff and the patient. However, this strategy of over-supply required the practice staff to continually click off the reminder throughout the day, which (especially in large practices) consumed quite a bit of the staff's research resources, leaving little time for the "real work" of the study, i.e. surveying patients. Moreover, this strategy of over-supply may have hindered or annoyed the practice staff while performing their daily tasks. In future, it will be necessary to adapt recruitment strategies by limiting the number of patients presented on screen per day and setting reachable, individual goals such as 15% recruitment of the practice's own net sample instead of a general goal of 200 patients per practice. Such refinements can be implemented cost-effectively with only a small amount of adjustment to the CTA tool.

With regards to selection bias, it should be no problem in future applications of the CTA tool to take early measures to counteract a recognised selection bias. For example, if the study centre (or an automatic analysis algorithm) detects a selection bias towards younger patients, the CTA tool could suppress the on-screen reminder for younger patients until the selection levels reflect the net sample.

Important primary care practice conditions like finding the time, the place and the personnel to immediately conduct a 10-minute survey within the practice setting most likely had a large influence on the number of study participants. In a further step, we will systematically analyse interviews with the participating practice staff about the comfort and efficiency of the recruitment software in daily practice. Especially the experiences of larger practices with the recruitment tool need to be critically reviewed, since this tool was not able to motivate the practice staff in larger practices to meet the recruitment target. Valuable user knowledge, combined with the research team's on-going technical and logistical evaluations, should then be incorporated into the further development of this technology.

## Conclusions

We have developed a CTA-based clinical trial tool that can be incorporated into future practice-based research endeavours requiring the participation of general practice patients. Although this technology is not yet ready for mass marketing, our intervention study has demonstrated potential benefits. Doctors and practice nurses were able to use this intervention to recruit and survey a large sample of general practice patients. The CTA tool described here eliminates the need for practice staff to screen patients in the waiting room since members of the target population are automatically recognised and communicated both to the local practice staff on their regular practice software screen as well as (pseudonymised) to the study centre via the Internet. Although the intervention did not prevent a selection bias while recruiting patients, the tool enabled researchers to recognise this bias so that some refinement would help to counteract a demographic selection bias in future projects. Further adaptations of this software can be applied in EPR-based recruitment strategies, e.g. for studies seeking to identify patients with specific conditions.

Studies requiring patient recruitment and data collection in busy general practices are dependent upon the willingness of both doctors and practice nurses to work together with the research team to find and survey the target sample. With this in mind, the further development of this technology should include not only technological refinements but also the input from the participating practice staff to create a strategy that is both technically efficient and suitable to practice conditions.

## Competing interests

SH, ST, SW, TS, CSN and WH have no conflicts of interest. MK is owner and manager of IT Choice, an IT company which developed and piloted the study's CTA tool and data bank.

## Authors' contributions

CSN and WH conceived and designed the study. MK designed and piloted the clinical trial alert (CTA) tool. ST collected data and was responsible for data management. SW and TS implemented the CTA tool and were responsible for quarterly updates, technical functioning of the tool and data management. SH and WH performed statistical analysis and interpreted data. SH drafted the manuscript. CSN, ST and SW commented extensively and contributed to the expansion of the text. SH and WH revised the manuscript. All authors read and approved the final manuscript. SH and WH are the guarantors.

## Pre-publication history

The pre-publication history for this paper can be accessed here:

http://www.biomedcentral.com/1471-2288/11/16/prepub
